# Crystal structure of 1-{1-[2-(phenyl­selan­yl)phen­yl]-1*H*-1,2,3-triazol-4-yl}cyclo­hexan-1-ol

**DOI:** 10.1107/S2056989015003242

**Published:** 2015-02-25

**Authors:** Leandro R. S. Camargo, Julio Zukerman-Schpector, Anna M. Deobald, Antonio L. Braga, Edward R. T. Tiekink

**Affiliations:** a Departmento de Química, Universidade Federal de São Carlos, 13565-905 São Carlos, SP, Brazil; b Departmento de Química, Universidade Federal de Santa Maria, 97105-900 Santa Maria, RS, Brazil; c Departmento de Química, Universidade Federal de Santa Catarina, 88040-900 Florianópolis, SC, Brazil; d Department of Chemistry, University of Malaya, 50603 Kuala Lumpur, Malaysia

**Keywords:** crystal structure, organoselenium, hydrogen bonding, Se⋯N halogen bonding

## Abstract

Two independent mol­ecules, *A* and *B*, comprise the asymmetric unit of the title compound, C_20_H_21_N_3_OSe. While the benzene ring directly bound to the central triazole ring is inclined to the same extent in both mol­ecules [dihedral angles = 40.41 (12) (mol­ecule *A*) and 44.14 (12)° (*B*)], greater differences are apparent in the dihedral angles between the Se-bound rings, *i.e.* 74.28 (12) (mol­ecule *A*) and 89.91 (11)° (*B*). Close intra­molecular Se⋯N inter­actions of 2.9311 (18) (mol­ecule *A*) and 2.9482 (18) Å (*B*) are noted. In the crystal, supra­molecular chains along the *a* axis are formed *via* O—H⋯N hydrogen bonding. These are connected into layers *via* C—H⋯O and C—H⋯N inter­actions; these stack along (01-1) without directional inter­molecular inter­actions between them.

## Related literature   

For background and synthesis of aryl­seleno-1,2,3-triazoles, including of the title compound, see: Deobald *et al.* (2011 ▸). For Se⋯N inter­actions, see: Pati & Zade (2014 ▸).
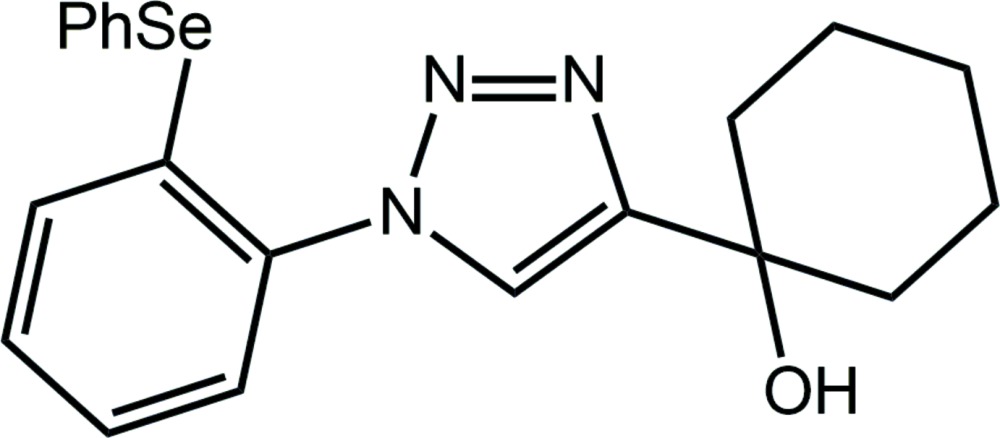


## Experimental   

### Crystal data  

C_20_H_21_N_3_OSe
*M* *_r_* = 398.36
Triclinic, 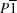

*a* = 10.7480 (4) Å
*b* = 13.7497 (6) Å
*c* = 13.8849 (5) Å
α = 112.432 (4)°
β = 92.889 (3)°
γ = 104.059 (3)°
*V* = 1816.28 (13) Å^3^
*Z* = 4
Cu *K*α radiation
μ = 2.89 mm^−1^
*T* = 100 K
0.20 × 0.10 × 0.05 mm

### Data collection  

Agilent SuperNova CCD diffractometer
Absorption correction: multi-scan (*CrysAlis PRO*; Agilent, 2011 ▸) *T*_min_ = 0.884, *T*_max_ = 1.000
26101 measured reflections
7291 independent reflections
7014 reflections with *I* > 2σ(*I*)
*R*_int_ = 0.087

### Refinement  

*R*[*F* ^2^ > 2σ(*F* ^2^)] = 0.039
*wR*(*F* ^2^) = 0.107
*S* = 1.08
7291 reflections
453 parameters
H-atom parameters constrained
Δρ_max_ = 1.06 e Å^−3^
Δρ_min_ = −1.11 e Å^−3^

### 

Data collection: *CrysAlis PRO* (Agilent, 2011 ▸); cell refinement: *CrysAlis PRO*; data reduction: *CrysAlis PRO*; program(s) used to solve structure: *SIR2014* (Burla *et al.*, 2015 ▸); program(s) used to refine structure: *SHELXL2014* (Sheldrick, 2015 ▸); molecular graphics: *ORTEP-3 for Windows* (Farrugia, 2012 ▸), *QMOL* (Gans & Shalloway, 2001 ▸) and *DIAMOND* (Brandenburg, 2006 ▸); software used to prepare material for publication: *MarvinSketch* (ChemAxon, 2010 ▸) and *publCIF* (Westrip, 2010 ▸).

## Supplementary Material

Crystal structure: contains datablock(s) I, New_Global_Publ_Block. DOI: 10.1107/S2056989015003242/hg5431sup1.cif

Structure factors: contains datablock(s) I. DOI: 10.1107/S2056989015003242/hg5431Isup2.hkl

Supporting information file. DOI: 10.1107/S2056989015003242/hg5431Isup3.cml

**Fig. 1.**
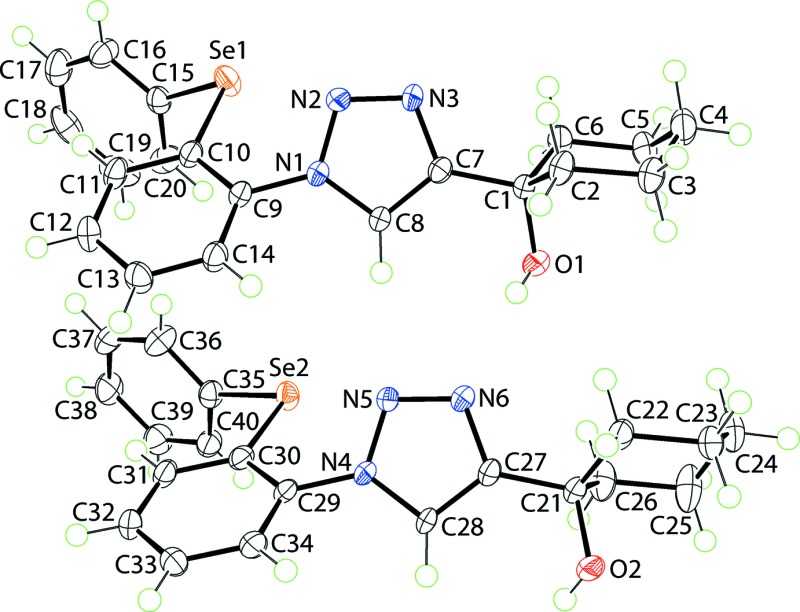
The molecular structures of the two independent molecules comprising the title compound showing the atom-labelling scheme and displacement ellipsoids at the 70% probability level.

. DOI: 10.1107/S2056989015003242/hg5431fig1.tif
The mol­ecular structures of the two independent mol­ecules comprising the title compound showing the atom-labelling scheme and displacement ellipsoids at the 70% probability level.

**Fig. 2.**
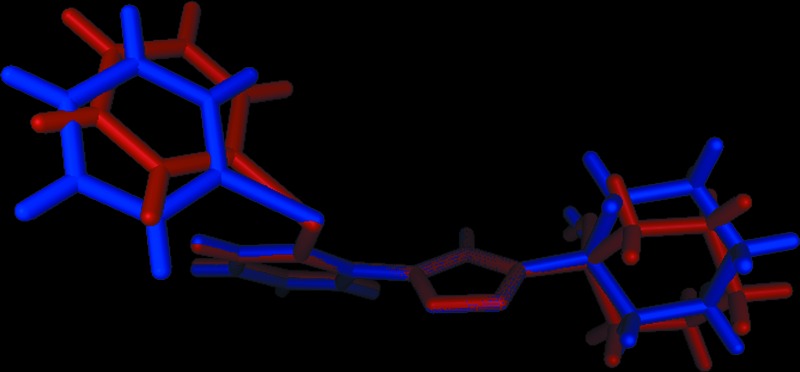
Superimposition of the two independent molecules. Molecule A is shown in red and B in blue. The molecules have been superimposed such that the triazol-4-yl rings are overlapped.

**Fig. 3.**
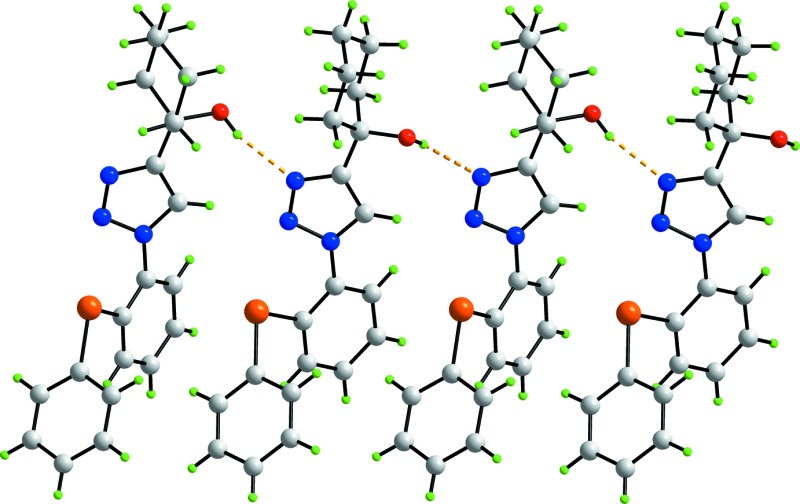
A view of the linear supramolecular sustained by O—H···N hydrogen bonds (orange dashed lines) and aligned along the a axis in the crystal packing.

**Fig. 4.**
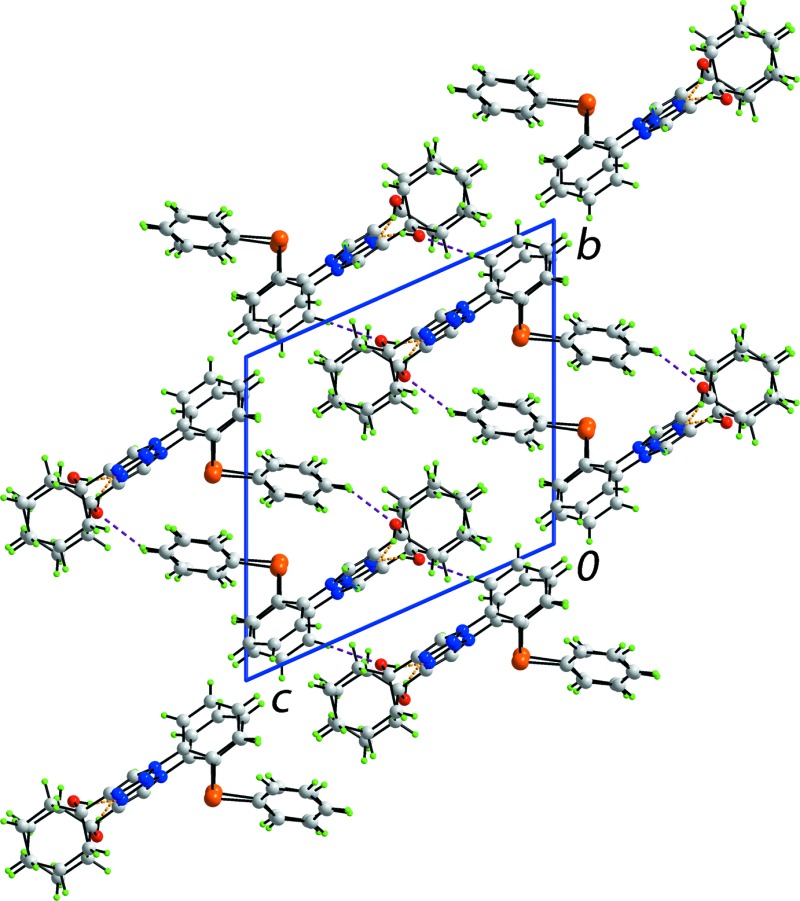
A view in projection down the a axis of the unit-cell contents. The O—H···N, C—H···O and C—H···N interactions are shown as orange, purple and blue dashed lines, respectively.

A B . DOI: 10.1107/S2056989015003242/hg5431fig2.tif
Superimposition of the two independent mol­ecules. Mol­ecule *A* is shown in red and *B* in blue. The mol­ecules have been superimposed such that the triazol-4-yl rings are overlapped.

a . DOI: 10.1107/S2056989015003242/hg5431fig3.tif
A view of the linear supra­molecular sustained by O—H⋯N hydrogen bonds (orange dashed lines) and aligned along the *a* axis in the crystal packing.

a . DOI: 10.1107/S2056989015003242/hg5431fig4.tif
A view in projection down the *a* axis of the unit-cell contents. The O—H⋯N, C—H⋯O and C—H⋯N inter­actions are shown as orange, purple and blue dashed lines, respectively.

CCDC reference: 1049508

Additional supporting information:  crystallographic information; 3D view; checkCIF report

## Figures and Tables

**Table 1:** Hydrogen-bond geometry (, )

*D*H*A*	*D*H	H*A*	*D* *A*	*D*H*A*
C8H8N5	0.95	2.32	3.229(3)	159
C18H18O1^i^	0.95	2.46	3.304(3)	148
C28H28N2^ii^	0.95	2.37	3.262(3)	157
C34H34O2^iii^	0.95	2.54	3.488(3)	173

Symmetry codes: (i) 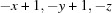; (ii) 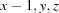; (iii) 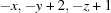.

## supplementary crystallographic information

### S1. Experimental

The compound was prepared in accord with the literature (Deobald *et al.*, 2011). Crystals were obtained by slow evaporation at room temperature from its methanol/dicloromethane (1:1 *v*/*v*) solution.

### S2. Refinement

Carbon-bound H-atoms were placed in calculated positions (C—H = 0.95 to 0.99 Å) and were included in the refinement in the riding model approximation, with *U_iso_*(H) = 1.25*U_eq_*(C). In the same way the O-bound H-atoms were constrained with O—H = 0.84 Å, with *U_iso_*(H) = 1.5*U_eq_*(O). The maximum and minimum residual electron density peaks of 1.06 and 1.11 e Å^-3^, respectively were located 0.98 Å and 0.84 Å from the Se2 and Se1 atoms, respectively.

### Crystal data

**Table:**

C_20_H_21_N_3_OSe	*Z* = 4
*M**_r_* = 398.36	*F*(000) = 816
Triclinic, *P*1	*D*_x_ = 1.457 Mg m^−^^3^
*a* = 10.7480 (4) Å	Cu *K*α radiation, λ = 1.54184 Å
*b* = 13.7497 (6) Å	Cell parameters from 19807 reflections
*c* = 13.8849 (5) Å	θ = 3.5–74.3°
α = 112.432 (4)°	µ = 2.89 mm^−^^1^
β = 92.889 (3)°	*T* = 100 K
γ = 104.059 (3)°	Prism, colourless
*V* = 1816.28 (13) Å^3^	0.20 × 0.10 × 0.05 mm

### Data collection

**Table:**

Agilent SuperNova CCD diffractometer	7014 reflections with *I* > 2σ(*I*)
Radiation source: SuperNova (Cu) X-ray Source	*R*_int_ = 0.087
ω scans	θ_max_ = 74.5°, θ_min_ = 3.5°
Absorption correction: multi-scan (*CrysAlis PRO*; Agilent, 2011)	*h* = −13→13
*T*_min_ = 0.884, *T*_max_ = 1.000	*k* = −17→17
26101 measured reflections	*l* = −17→16
7291 independent reflections	

### Refinement

**Table:**

Refinement on *F*^2^	0 restraints
Least-squares matrix: full	Hydrogen site location: inferred from neighbouring sites
*R*[*F*^2^ > 2σ(*F*^2^)] = 0.039	H-atom parameters constrained
*wR*(*F*^2^) = 0.107	*w* = 1/[σ^2^(*F*_o_^2^) + (0.0551*P*)^2^ + 1.3888*P*] where *P* = (*F*_o_^2^ + 2*F*_c_^2^)/3
*S* = 1.08	(Δ/σ)_max_ < 0.001
7291 reflections	Δρ_max_ = 1.06 e Å^−^^3^
453 parameters	Δρ_min_ = −1.11 e Å^−^^3^

### Special details

**Table:**

Geometry. All e.s.d.'s (except the e.s.d. in the dihedral angle between two l.s. planes) are estimated using the full covariance matrix. The cell e.s.d.'s are taken into account individually in the estimation of e.s.d.'s in distances, angles and torsion angles; correlations between e.s.d.'s in cell parameters are only used when they are defined by crystal symmetry. An approximate (isotropic) treatment of cell e.s.d.'s is used for estimating e.s.d.'s involving l.s. planes.

### Fractional atomic coordinates and isotropic or equivalent isotropic displacement parameters (Å^2^)

**Table:**

	*x*	*y*	*z*	*U*_iso_*/*U*_eq_	
Se1	0.79205 (2)	0.69748 (2)	0.10496 (2)	0.01986 (9)	
O1	0.42441 (13)	0.72340 (12)	0.48557 (12)	0.0183 (3)	
H1O	0.3939	0.7718	0.4792	0.027*	
N1	0.64154 (16)	0.81972 (13)	0.27686 (13)	0.0133 (3)	
N2	0.75895 (16)	0.82424 (14)	0.32115 (14)	0.0161 (4)	
N3	0.74173 (17)	0.80356 (15)	0.40452 (14)	0.0155 (3)	
C1	0.56313 (18)	0.76209 (16)	0.50571 (15)	0.0133 (4)	
C2	0.6108 (2)	0.86650 (17)	0.60809 (16)	0.0177 (4)	
H2A	0.7062	0.8956	0.6162	0.021*	
H2B	0.5715	0.9228	0.6030	0.021*	
C3	0.5763 (2)	0.84618 (18)	0.70586 (17)	0.0205 (4)	
H3A	0.6154	0.9142	0.7700	0.025*	
H3B	0.4808	0.8272	0.7028	0.025*	
C4	0.6255 (2)	0.75324 (18)	0.71257 (18)	0.0228 (5)	
H4A	0.5977	0.7390	0.7740	0.027*	
H4B	0.7217	0.7753	0.7229	0.027*	
C5	0.5723 (2)	0.64827 (18)	0.61151 (17)	0.0207 (4)	
H5A	0.6074	0.5897	0.6163	0.025*	
H5B	0.4764	0.6231	0.6040	0.025*	
C6	0.6101 (2)	0.66891 (16)	0.51505 (16)	0.0163 (4)	
H6A	0.5722	0.6009	0.4506	0.020*	
H6B	0.7058	0.6876	0.5199	0.020*	
C7	0.61347 (19)	0.78607 (15)	0.41545 (15)	0.0131 (4)	
C8	0.54837 (18)	0.79664 (15)	0.33409 (15)	0.0131 (4)	
H8	0.4583	0.7895	0.3207	0.016*	
C9	0.62903 (18)	0.84138 (16)	0.18508 (16)	0.0135 (4)	
C10	0.69676 (19)	0.79898 (16)	0.10285 (16)	0.0143 (4)	
C11	0.6872 (2)	0.82657 (17)	0.01671 (17)	0.0178 (4)	
H11	0.7361	0.8019	−0.0382	0.021*	
C12	0.6071 (2)	0.88957 (17)	0.01020 (17)	0.0189 (4)	
H12	0.6005	0.9071	−0.0494	0.023*	
C13	0.5359 (2)	0.92755 (17)	0.09105 (17)	0.0190 (4)	
H13	0.4792	0.9692	0.0856	0.023*	
C14	0.5482 (2)	0.90447 (16)	0.17880 (16)	0.0169 (4)	
H14	0.5016	0.9316	0.2347	0.020*	
C15	0.7598 (2)	0.61407 (16)	−0.04505 (17)	0.0164 (4)	
C16	0.8578 (2)	0.62498 (18)	−0.10508 (18)	0.0215 (4)	
H16	0.9411	0.6749	−0.0721	0.026*	
C17	0.8337 (3)	0.5628 (2)	−0.2132 (2)	0.0277 (5)	
H17	0.9011	0.5702	−0.2538	0.033*	
C18	0.7131 (3)	0.49049 (19)	−0.26226 (19)	0.0286 (5)	
H18	0.6973	0.4479	−0.3363	0.034*	
C19	0.6151 (2)	0.48054 (18)	−0.2025 (2)	0.0290 (6)	
H19	0.5314	0.4317	−0.2360	0.035*	
C20	0.6382 (2)	0.54102 (18)	−0.09454 (19)	0.0230 (5)	
H20	0.5708	0.5327	−0.0541	0.028*	
Se2	0.23728 (2)	0.68144 (2)	0.10894 (2)	0.01583 (8)	
O2	−0.02727 (14)	0.86348 (14)	0.56157 (12)	0.0220 (3)	
H2O	−0.0859	0.8335	0.5085	0.033*	
N4	0.15650 (16)	0.85609 (13)	0.29668 (13)	0.0135 (3)	
N5	0.26503 (16)	0.83739 (15)	0.33016 (14)	0.0169 (4)	
N6	0.24879 (17)	0.82511 (15)	0.41776 (15)	0.0172 (4)	
C21	0.08158 (19)	0.82042 (17)	0.53679 (16)	0.0155 (4)	
C22	0.1860 (2)	0.88455 (17)	0.63518 (16)	0.0171 (4)	
H22A	0.2685	0.8672	0.6183	0.021*	
H22B	0.2007	0.9641	0.6550	0.021*	
C23	0.1488 (2)	0.85807 (18)	0.72899 (17)	0.0204 (4)	
H23A	0.2214	0.8980	0.7889	0.025*	
H23B	0.0720	0.8833	0.7514	0.025*	
C24	0.1178 (2)	0.7356 (2)	0.70098 (19)	0.0267 (5)	
H24A	0.0915	0.7205	0.7624	0.032*	
H24B	0.1963	0.7111	0.6835	0.032*	
C25	0.0081 (3)	0.6720 (2)	0.60657 (18)	0.0300 (6)	
H25A	−0.0093	0.5923	0.5876	0.036*	
H25B	−0.0722	0.6926	0.6259	0.036*	
C26	0.0444 (2)	0.69690 (18)	0.51152 (17)	0.0233 (5)	
H26A	−0.0301	0.6583	0.4531	0.028*	
H26B	0.1184	0.6682	0.4873	0.028*	
C27	0.13023 (19)	0.83756 (16)	0.44306 (15)	0.0142 (4)	
C28	0.07058 (19)	0.85768 (16)	0.36567 (15)	0.0146 (4)	
H28	−0.0123	0.8700	0.3612	0.017*	
C29	0.14779 (18)	0.87469 (16)	0.20299 (16)	0.0137 (4)	
C30	0.18517 (18)	0.80731 (16)	0.11135 (16)	0.0140 (4)	
C31	0.18131 (19)	0.83302 (17)	0.02395 (17)	0.0165 (4)	
H31	0.2096	0.7903	−0.0379	0.020*	
C32	0.1367 (2)	0.92036 (17)	0.02572 (17)	0.0177 (4)	
H32	0.1346	0.9369	−0.0347	0.021*	
C33	0.0950 (2)	0.98361 (17)	0.11573 (17)	0.0185 (4)	
H33	0.0626	1.0421	0.1161	0.022*	
C34	0.10072 (19)	0.96140 (17)	0.20476 (16)	0.0163 (4)	
H34	0.0729	1.0047	0.2665	0.020*	
C35	0.2304 (2)	0.60755 (16)	−0.04120 (17)	0.0161 (4)	
C36	0.3411 (2)	0.62954 (18)	−0.08591 (19)	0.0230 (5)	
H36	0.4187	0.6821	−0.0428	0.028*	
C37	0.3381 (2)	0.5748 (2)	−0.19308 (19)	0.0251 (5)	
H37	0.4135	0.5906	−0.2235	0.030*	
C38	0.2256 (2)	0.49665 (19)	−0.25669 (18)	0.0241 (5)	
H38	0.2239	0.4594	−0.3303	0.029*	
C39	0.1154 (2)	0.47333 (18)	−0.21178 (18)	0.0223 (5)	
H39	0.0386	0.4195	−0.2548	0.027*	
C40	0.1176 (2)	0.52865 (17)	−0.10406 (17)	0.0181 (4)	
H40	0.0423	0.5126	−0.0735	0.022*	

### Atomic displacement parameters (Å^2^)

**Table:**

	*U*^11^	*U*^22^	*U*^33^	*U*^12^	*U*^13^	*U*^23^
Se1	0.02619 (14)	0.02673 (14)	0.01324 (14)	0.01777 (10)	0.00654 (10)	0.00845 (10)
O1	0.0129 (7)	0.0239 (7)	0.0216 (8)	0.0041 (6)	0.0051 (6)	0.0134 (6)
N1	0.0133 (8)	0.0166 (8)	0.0119 (8)	0.0052 (6)	0.0047 (6)	0.0069 (6)
N2	0.0119 (8)	0.0250 (9)	0.0134 (9)	0.0061 (7)	0.0033 (6)	0.0092 (7)
N3	0.0154 (8)	0.0227 (8)	0.0124 (8)	0.0073 (7)	0.0060 (7)	0.0099 (7)
C1	0.0118 (9)	0.0176 (9)	0.0112 (9)	0.0034 (7)	0.0028 (7)	0.0070 (8)
C2	0.0222 (10)	0.0174 (9)	0.0135 (10)	0.0061 (8)	0.0079 (8)	0.0056 (8)
C3	0.0247 (10)	0.0231 (10)	0.0137 (10)	0.0074 (8)	0.0067 (8)	0.0067 (8)
C4	0.0268 (11)	0.0272 (11)	0.0161 (11)	0.0057 (9)	0.0043 (9)	0.0116 (9)
C5	0.0261 (11)	0.0217 (10)	0.0180 (11)	0.0060 (8)	0.0039 (9)	0.0125 (9)
C6	0.0203 (10)	0.0167 (9)	0.0146 (10)	0.0072 (8)	0.0049 (8)	0.0077 (8)
C7	0.0141 (9)	0.0132 (8)	0.0115 (9)	0.0044 (7)	0.0042 (7)	0.0040 (7)
C8	0.0125 (8)	0.0167 (9)	0.0120 (9)	0.0044 (7)	0.0040 (7)	0.0075 (7)
C9	0.0137 (9)	0.0145 (8)	0.0137 (10)	0.0034 (7)	0.0046 (7)	0.0072 (7)
C10	0.0154 (9)	0.0147 (9)	0.0154 (10)	0.0056 (7)	0.0062 (8)	0.0078 (7)
C11	0.0217 (10)	0.0199 (10)	0.0150 (10)	0.0080 (8)	0.0099 (8)	0.0083 (8)
C12	0.0266 (11)	0.0186 (9)	0.0142 (10)	0.0065 (8)	0.0053 (8)	0.0091 (8)
C13	0.0240 (10)	0.0193 (9)	0.0201 (11)	0.0121 (8)	0.0066 (9)	0.0109 (8)
C14	0.0195 (10)	0.0189 (9)	0.0152 (10)	0.0077 (8)	0.0070 (8)	0.0083 (8)
C15	0.0202 (10)	0.0164 (9)	0.0163 (10)	0.0087 (8)	0.0047 (8)	0.0082 (8)
C16	0.0198 (10)	0.0245 (10)	0.0198 (11)	0.0067 (8)	0.0079 (9)	0.0078 (9)
C17	0.0369 (13)	0.0317 (12)	0.0225 (12)	0.0165 (10)	0.0149 (10)	0.0142 (10)
C18	0.0481 (15)	0.0245 (11)	0.0135 (11)	0.0198 (11)	−0.0015 (10)	0.0030 (9)
C19	0.0266 (12)	0.0167 (10)	0.0354 (14)	0.0042 (9)	−0.0078 (10)	0.0046 (10)
C20	0.0185 (10)	0.0212 (10)	0.0304 (13)	0.0056 (8)	0.0072 (9)	0.0114 (9)
Se2	0.02069 (13)	0.01657 (13)	0.01253 (14)	0.00764 (9)	0.00494 (9)	0.00664 (9)
O2	0.0158 (7)	0.0399 (9)	0.0138 (7)	0.0139 (6)	0.0063 (6)	0.0106 (6)
N4	0.0128 (7)	0.0188 (8)	0.0128 (8)	0.0072 (6)	0.0065 (6)	0.0082 (7)
N5	0.0136 (8)	0.0296 (9)	0.0116 (8)	0.0088 (7)	0.0044 (7)	0.0108 (7)
N6	0.0157 (8)	0.0265 (9)	0.0144 (9)	0.0100 (7)	0.0071 (7)	0.0107 (7)
C21	0.0138 (9)	0.0221 (10)	0.0121 (9)	0.0062 (8)	0.0054 (7)	0.0075 (8)
C22	0.0174 (9)	0.0208 (10)	0.0131 (10)	0.0049 (8)	0.0049 (8)	0.0068 (8)
C23	0.0222 (10)	0.0280 (11)	0.0116 (10)	0.0085 (9)	0.0040 (8)	0.0077 (8)
C24	0.0339 (13)	0.0313 (12)	0.0201 (12)	0.0079 (10)	0.0066 (10)	0.0167 (10)
C25	0.0413 (14)	0.0263 (11)	0.0189 (12)	−0.0009 (10)	0.0073 (11)	0.0117 (9)
C26	0.0294 (11)	0.0206 (10)	0.0144 (10)	−0.0005 (9)	0.0036 (9)	0.0058 (8)
C27	0.0148 (9)	0.0177 (9)	0.0112 (9)	0.0055 (7)	0.0055 (7)	0.0060 (7)
C28	0.0136 (9)	0.0219 (9)	0.0115 (9)	0.0068 (7)	0.0069 (7)	0.0088 (8)
C29	0.0101 (8)	0.0186 (9)	0.0138 (10)	0.0033 (7)	0.0047 (7)	0.0080 (8)
C30	0.0109 (8)	0.0159 (9)	0.0139 (10)	0.0022 (7)	0.0029 (7)	0.0058 (8)
C31	0.0158 (9)	0.0208 (10)	0.0143 (10)	0.0048 (8)	0.0065 (8)	0.0085 (8)
C32	0.0181 (10)	0.0236 (10)	0.0148 (10)	0.0056 (8)	0.0047 (8)	0.0114 (8)
C33	0.0200 (10)	0.0240 (10)	0.0176 (10)	0.0112 (8)	0.0054 (8)	0.0115 (8)
C34	0.0160 (9)	0.0203 (9)	0.0146 (10)	0.0080 (8)	0.0054 (8)	0.0070 (8)
C35	0.0199 (10)	0.0151 (9)	0.0151 (10)	0.0072 (8)	0.0058 (8)	0.0065 (8)
C36	0.0206 (10)	0.0231 (10)	0.0240 (12)	0.0057 (8)	0.0096 (9)	0.0078 (9)
C37	0.0221 (11)	0.0336 (12)	0.0230 (12)	0.0103 (9)	0.0127 (9)	0.0123 (10)
C38	0.0309 (12)	0.0294 (11)	0.0133 (10)	0.0136 (10)	0.0085 (9)	0.0063 (9)
C39	0.0242 (11)	0.0222 (10)	0.0184 (11)	0.0066 (9)	0.0037 (9)	0.0061 (9)
C40	0.0180 (10)	0.0199 (10)	0.0166 (10)	0.0046 (8)	0.0046 (8)	0.0080 (8)

### Geometric parameters (Å, º)

**Table:**

Se1—C15	1.920 (2)	Se2—C35	1.927 (2)
Se1—C10	1.929 (2)	Se2—C30	1.934 (2)
O1—C1	1.429 (2)	O2—C21	1.435 (2)
O1—H1O	0.8400	O2—H2O	0.8400
N1—N2	1.352 (2)	N4—N5	1.351 (2)
N1—C8	1.361 (2)	N4—C28	1.361 (2)
N1—C9	1.422 (3)	N4—C29	1.421 (3)
N2—N3	1.305 (3)	N5—N6	1.305 (3)
N3—C7	1.367 (3)	N6—C27	1.372 (3)
C1—C7	1.506 (3)	C21—C27	1.505 (3)
C1—C6	1.532 (3)	C21—C22	1.533 (3)
C1—C2	1.535 (3)	C21—C26	1.539 (3)
C2—C3	1.532 (3)	C22—C23	1.527 (3)
C2—H2A	0.9900	C22—H22A	0.9900
C2—H2B	0.9900	C22—H22B	0.9900
C3—C4	1.528 (3)	C23—C24	1.521 (3)
C3—H3A	0.9900	C23—H23A	0.9900
C3—H3B	0.9900	C23—H23B	0.9900
C4—C5	1.534 (3)	C24—C25	1.531 (3)
C4—H4A	0.9900	C24—H24A	0.9900
C4—H4B	0.9900	C24—H24B	0.9900
C5—C6	1.526 (3)	C25—C26	1.531 (3)
C5—H5A	0.9900	C25—H25A	0.9900
C5—H5B	0.9900	C25—H25B	0.9900
C6—H6A	0.9900	C26—H26A	0.9900
C6—H6B	0.9900	C26—H26B	0.9900
C7—C8	1.372 (3)	C27—C28	1.373 (3)
C8—H8	0.9500	C28—H28	0.9500
C9—C14	1.390 (3)	C29—C34	1.396 (3)
C9—C10	1.398 (3)	C29—C30	1.404 (3)
C10—C11	1.392 (3)	C30—C31	1.389 (3)
C11—C12	1.383 (3)	C31—C32	1.390 (3)
C11—H11	0.9500	C31—H31	0.9500
C12—C13	1.397 (3)	C32—C33	1.391 (3)
C12—H12	0.9500	C32—H32	0.9500
C13—C14	1.379 (3)	C33—C34	1.384 (3)
C13—H13	0.9500	C33—H33	0.9500
C14—H14	0.9500	C34—H34	0.9500
C15—C20	1.391 (3)	C35—C36	1.392 (3)
C15—C16	1.391 (3)	C35—C40	1.393 (3)
C16—C17	1.387 (3)	C36—C37	1.382 (3)
C16—H16	0.9500	C36—H36	0.9500
C17—C18	1.379 (4)	C37—C38	1.390 (3)
C17—H17	0.9500	C37—H37	0.9500
C18—C19	1.386 (4)	C38—C39	1.391 (3)
C18—H18	0.9500	C38—H38	0.9500
C19—C20	1.382 (4)	C39—C40	1.391 (3)
C19—H19	0.9500	C39—H39	0.9500
C20—H20	0.9500	C40—H40	0.9500
			
C15—Se1—C10	95.64 (9)	C35—Se2—C30	98.68 (9)
C1—O1—H1O	109.5	C21—O2—H2O	109.5
N2—N1—C8	110.56 (16)	N5—N4—C28	110.57 (17)
N2—N1—C9	120.58 (16)	N5—N4—C29	119.73 (16)
C8—N1—C9	128.83 (17)	C28—N4—C29	129.65 (17)
N3—N2—N1	107.20 (16)	N6—N5—N4	107.29 (16)
N2—N3—C7	109.58 (17)	N5—N6—C27	109.59 (17)
O1—C1—C7	110.33 (16)	O2—C21—C27	110.34 (17)
O1—C1—C6	105.90 (16)	O2—C21—C22	105.70 (16)
C7—C1—C6	109.86 (16)	C27—C21—C22	110.48 (16)
O1—C1—C2	110.90 (16)	O2—C21—C26	111.10 (17)
C7—C1—C2	109.18 (16)	C27—C21—C26	108.70 (16)
C6—C1—C2	110.64 (17)	C22—C21—C26	110.50 (18)
C3—C2—C1	112.56 (17)	C23—C22—C21	112.61 (17)
C3—C2—H2A	109.1	C23—C22—H22A	109.1
C1—C2—H2A	109.1	C21—C22—H22A	109.1
C3—C2—H2B	109.1	C23—C22—H22B	109.1
C1—C2—H2B	109.1	C21—C22—H22B	109.1
H2A—C2—H2B	107.8	H22A—C22—H22B	107.8
C4—C3—C2	111.17 (17)	C24—C23—C22	111.29 (17)
C4—C3—H3A	109.4	C24—C23—H23A	109.4
C2—C3—H3A	109.4	C22—C23—H23A	109.4
C4—C3—H3B	109.4	C24—C23—H23B	109.4
C2—C3—H3B	109.4	C22—C23—H23B	109.4
H3A—C3—H3B	108.0	H23A—C23—H23B	108.0
C3—C4—C5	110.64 (19)	C23—C24—C25	110.2 (2)
C3—C4—H4A	109.5	C23—C24—H24A	109.6
C5—C4—H4A	109.5	C25—C24—H24A	109.6
C3—C4—H4B	109.5	C23—C24—H24B	109.6
C5—C4—H4B	109.5	C25—C24—H24B	109.6
H4A—C4—H4B	108.1	H24A—C24—H24B	108.1
C6—C5—C4	110.60 (18)	C26—C25—C24	110.7 (2)
C6—C5—H5A	109.5	C26—C25—H25A	109.5
C4—C5—H5A	109.5	C24—C25—H25A	109.5
C6—C5—H5B	109.5	C26—C25—H25B	109.5
C4—C5—H5B	109.5	C24—C25—H25B	109.5
H5A—C5—H5B	108.1	H25A—C25—H25B	108.1
C5—C6—C1	112.04 (16)	C25—C26—C21	112.70 (18)
C5—C6—H6A	109.2	C25—C26—H26A	109.1
C1—C6—H6A	109.2	C21—C26—H26A	109.1
C5—C6—H6B	109.2	C25—C26—H26B	109.1
C1—C6—H6B	109.2	C21—C26—H26B	109.1
H6A—C6—H6B	107.9	H26A—C26—H26B	107.8
N3—C7—C8	108.03 (18)	N6—C27—C28	107.76 (17)
N3—C7—C1	122.24 (18)	N6—C27—C21	121.25 (18)
C8—C7—C1	129.71 (18)	C28—C27—C21	130.79 (18)
N1—C8—C7	104.62 (17)	N4—C28—C27	104.77 (17)
N1—C8—H8	127.7	N4—C28—H28	127.6
C7—C8—H8	127.7	C27—C28—H28	127.6
C14—C9—C10	121.08 (19)	C34—C29—C30	121.17 (19)
C14—C9—N1	118.83 (17)	C34—C29—N4	118.11 (17)
C10—C9—N1	120.09 (18)	C30—C29—N4	120.71 (18)
C11—C10—C9	118.36 (19)	C31—C30—C29	118.17 (19)
C11—C10—Se1	120.90 (15)	C31—C30—Se2	121.99 (15)
C9—C10—Se1	120.62 (16)	C29—C30—Se2	119.83 (16)
C12—C11—C10	120.68 (19)	C30—C31—C32	120.90 (19)
C12—C11—H11	119.7	C30—C31—H31	119.5
C10—C11—H11	119.7	C32—C31—H31	119.5
C11—C12—C13	120.2 (2)	C31—C32—C33	120.2 (2)
C11—C12—H12	119.9	C31—C32—H32	119.9
C13—C12—H12	119.9	C33—C32—H32	119.9
C14—C13—C12	119.8 (2)	C34—C33—C32	120.1 (2)
C14—C13—H13	120.1	C34—C33—H33	120.0
C12—C13—H13	120.1	C32—C33—H33	120.0
C13—C14—C9	119.76 (19)	C33—C34—C29	119.39 (18)
C13—C14—H14	120.1	C33—C34—H34	120.3
C9—C14—H14	120.1	C29—C34—H34	120.3
C20—C15—C16	119.4 (2)	C36—C35—C40	119.97 (19)
C20—C15—Se1	120.11 (16)	C36—C35—Se2	119.49 (16)
C16—C15—Se1	120.51 (16)	C40—C35—Se2	120.46 (15)
C17—C16—C15	119.9 (2)	C37—C36—C35	119.9 (2)
C17—C16—H16	120.1	C37—C36—H36	120.1
C15—C16—H16	120.1	C35—C36—H36	120.1
C18—C17—C16	120.7 (2)	C36—C37—C38	120.6 (2)
C18—C17—H17	119.6	C36—C37—H37	119.7
C16—C17—H17	119.6	C38—C37—H37	119.7
C17—C18—C19	119.3 (2)	C37—C38—C39	119.6 (2)
C17—C18—H18	120.3	C37—C38—H38	120.2
C19—C18—H18	120.3	C39—C38—H38	120.2
C20—C19—C18	120.6 (2)	C38—C39—C40	120.1 (2)
C20—C19—H19	119.7	C38—C39—H39	119.9
C18—C19—H19	119.7	C40—C39—H39	119.9
C19—C20—C15	120.1 (2)	C39—C40—C35	119.8 (2)
C19—C20—H20	119.9	C39—C40—H40	120.1
C15—C20—H20	119.9	C35—C40—H40	120.1
			
C8—N1—N2—N3	0.6 (2)	C28—N4—N5—N6	1.1 (2)
C9—N1—N2—N3	178.54 (16)	C29—N4—N5—N6	178.85 (17)
N1—N2—N3—C7	−0.3 (2)	N4—N5—N6—C27	−1.0 (2)
O1—C1—C2—C3	64.5 (2)	O2—C21—C22—C23	68.3 (2)
C7—C1—C2—C3	−173.77 (17)	C27—C21—C22—C23	−172.32 (17)
C6—C1—C2—C3	−52.7 (2)	C26—C21—C22—C23	−52.0 (2)
C1—C2—C3—C4	54.3 (2)	C21—C22—C23—C24	55.7 (2)
C2—C3—C4—C5	−56.0 (2)	C22—C23—C24—C25	−57.6 (2)
C3—C4—C5—C6	57.4 (2)	C23—C24—C25—C26	57.4 (3)
C4—C5—C6—C1	−56.9 (2)	C24—C25—C26—C21	−55.4 (3)
O1—C1—C6—C5	−66.1 (2)	O2—C21—C26—C25	−64.8 (2)
C7—C1—C6—C5	174.77 (16)	C27—C21—C26—C25	173.55 (19)
C2—C1—C6—C5	54.2 (2)	C22—C21—C26—C25	52.2 (2)
N2—N3—C7—C8	0.0 (2)	N5—N6—C27—C28	0.5 (2)
N2—N3—C7—C1	−178.91 (17)	N5—N6—C27—C21	175.98 (18)
O1—C1—C7—N3	−167.04 (17)	O2—C21—C27—N6	165.99 (17)
C6—C1—C7—N3	−50.6 (2)	C22—C21—C27—N6	49.5 (2)
C2—C1—C7—N3	70.8 (2)	C26—C21—C27—N6	−71.9 (2)
O1—C1—C7—C8	14.3 (3)	O2—C21—C27—C28	−19.7 (3)
C6—C1—C7—C8	130.7 (2)	C22—C21—C27—C28	−136.3 (2)
C2—C1—C7—C8	−107.8 (2)	C26—C21—C27—C28	102.3 (2)
N2—N1—C8—C7	−0.5 (2)	N5—N4—C28—C27	−0.8 (2)
C9—N1—C8—C7	−178.31 (17)	C29—N4—C28—C27	−178.21 (18)
N3—C7—C8—N1	0.3 (2)	N6—C27—C28—N4	0.2 (2)
C1—C7—C8—N1	179.13 (18)	C21—C27—C28—N4	−174.70 (19)
N2—N1—C9—C14	−138.21 (19)	N5—N4—C29—C34	−134.48 (19)
C8—N1—C9—C14	39.4 (3)	C28—N4—C29—C34	42.7 (3)
N2—N1—C9—C10	42.2 (3)	N5—N4—C29—C30	45.7 (3)
C8—N1—C9—C10	−140.3 (2)	C28—N4—C29—C30	−137.1 (2)
C14—C9—C10—C11	3.6 (3)	C34—C29—C30—C31	3.7 (3)
N1—C9—C10—C11	−176.80 (17)	N4—C29—C30—C31	−176.53 (17)
C14—C9—C10—Se1	−172.58 (15)	C34—C29—C30—Se2	−175.30 (15)
N1—C9—C10—Se1	7.0 (3)	N4—C29—C30—Se2	4.5 (2)
C9—C10—C11—C12	−3.4 (3)	C29—C30—C31—C32	−2.6 (3)
Se1—C10—C11—C12	172.75 (16)	Se2—C30—C31—C32	176.40 (15)
C10—C11—C12—C13	0.8 (3)	C30—C31—C32—C33	0.0 (3)
C11—C12—C13—C14	1.7 (3)	C31—C32—C33—C34	1.5 (3)
C12—C13—C14—C9	−1.5 (3)	C32—C33—C34—C29	−0.4 (3)
C10—C9—C14—C13	−1.2 (3)	C30—C29—C34—C33	−2.2 (3)
N1—C9—C14—C13	179.22 (18)	N4—C29—C34—C33	177.99 (18)
C20—C15—C16—C17	−0.3 (3)	C40—C35—C36—C37	1.4 (3)
Se1—C15—C16—C17	178.92 (18)	Se2—C35—C36—C37	178.26 (18)
C15—C16—C17—C18	0.4 (4)	C35—C36—C37—C38	−0.8 (4)
C16—C17—C18—C19	0.2 (4)	C36—C37—C38—C39	−0.2 (4)
C17—C18—C19—C20	−1.0 (4)	C37—C38—C39—C40	0.6 (4)
C18—C19—C20—C15	1.1 (4)	C38—C39—C40—C35	0.0 (3)
C16—C15—C20—C19	−0.4 (3)	C36—C35—C40—C39	−1.1 (3)
Se1—C15—C20—C19	−179.68 (18)	Se2—C35—C40—C39	−177.84 (17)

### Hydrogen-bond geometry (Å, º)

**Table:**

*D*—H···*A*	*D*—H	H···*A*	*D*···*A*	*D*—H···*A*
C8—H8···N5	0.95	2.32	3.229 (3)	159
C18—H18···O1^i^	0.95	2.46	3.304 (3)	148
C28—H28···N2^ii^	0.95	2.37	3.262 (3)	157
C34—H34···O2^iii^	0.95	2.54	3.488 (3)	173

Symmetry codes: (i) −*x*+1, −*y*+1, −*z*; (ii) *x*−1, *y*, *z*; (iii) −*x*, −*y*+2, −*z*+1.
