# Supplementary figures and images for: Crystal structure of 1-{1-[2-(phenyl­selan­yl)phen­yl]-1H-1,2,3-triazol-4-yl}cyclo­hexan-1-ol
Source: Acta Crystallogr E Crystallogr Commun. 2015 Feb 25;71(Pt 3):o200–1. doi: 10.1107/S2056989015003242 (PMC4350684; doi:10.1107/S2056989015003242)

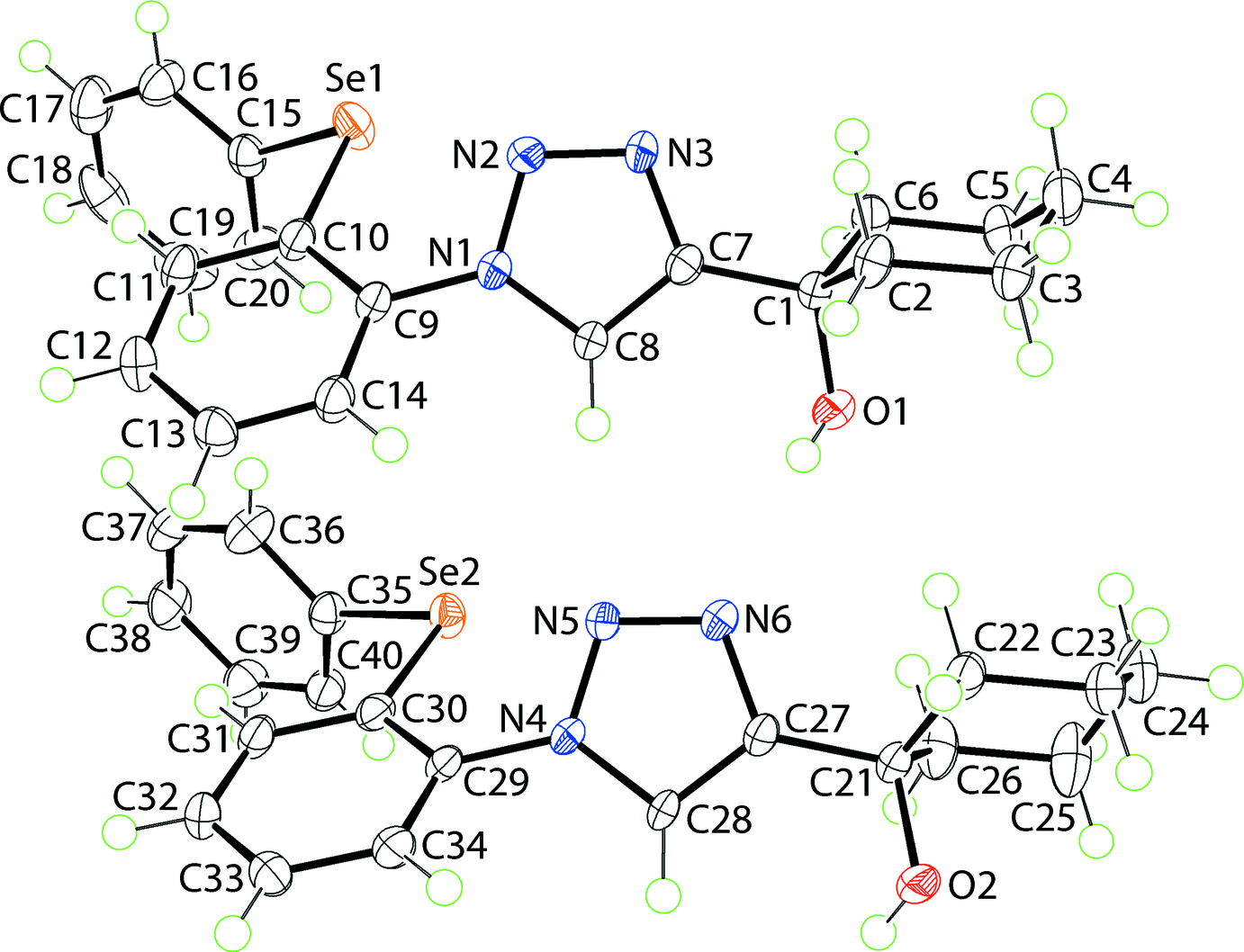

Supplement: Supplementary file 4 [file e-71-0o200-fig1.tif]

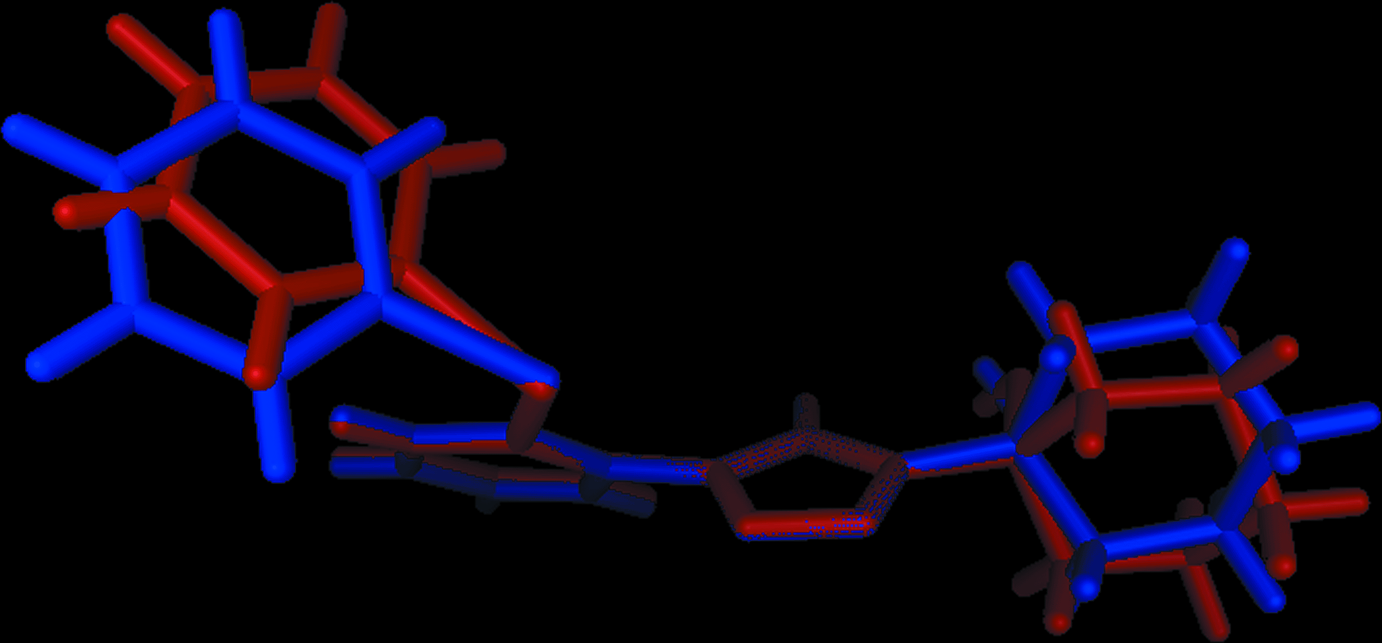

Supplement: Supplementary file 5 [file e-71-0o200-fig2.tif]

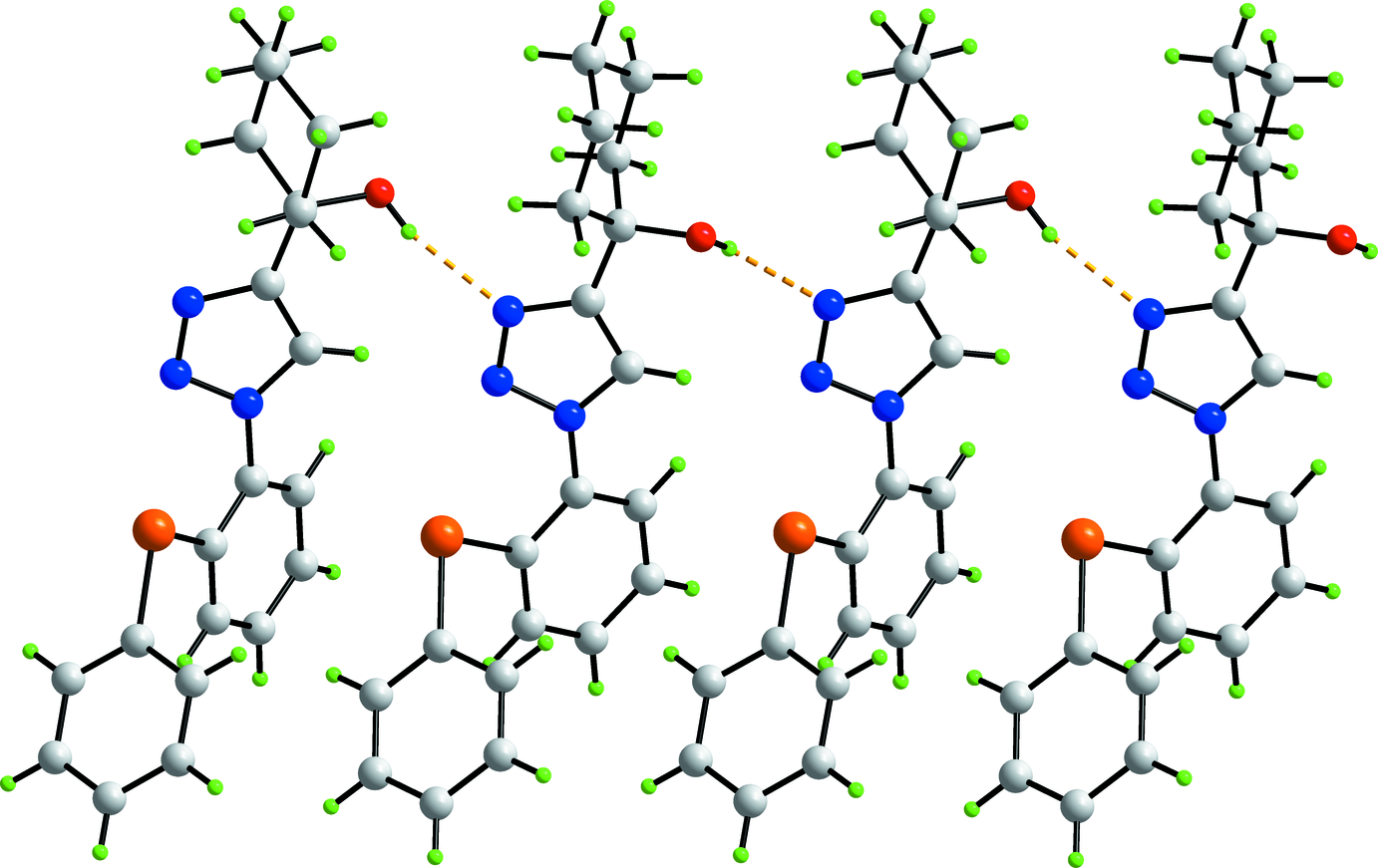

Supplement: Supplementary file 6 [file e-71-0o200-fig3.tif]

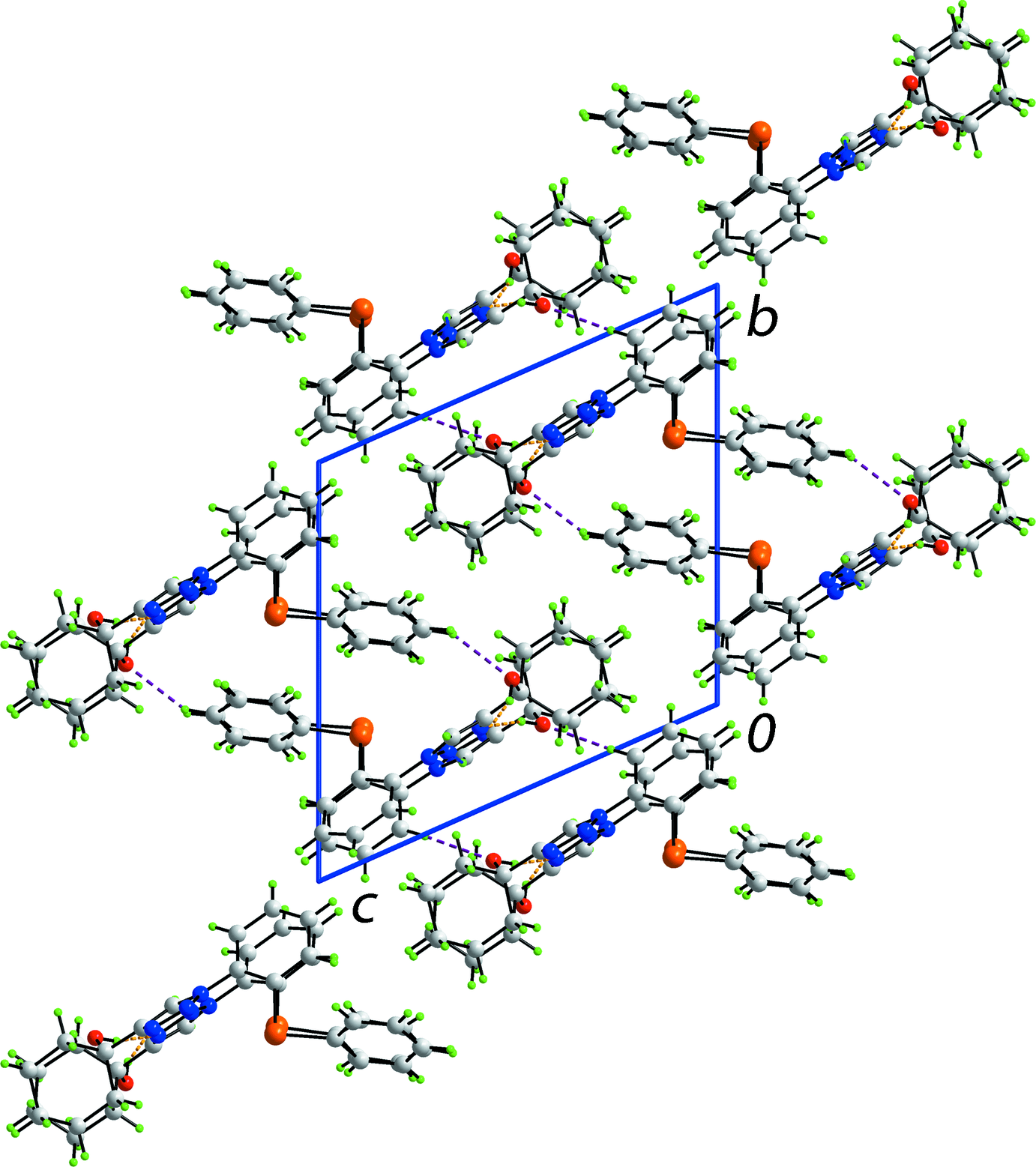

Supplement: Supplementary file 7 [file e-71-0o200-fig4.tif]
